# COVID‐19 Vaccine‐Induced Severe Pneumonitis

**DOI:** 10.1002/rcr2.70274

**Published:** 2025-08-21

**Authors:** Tatsuro Suzuki, Hiromi Furuta, Misa Naganawa, Keiko Hayashi, Hiroko Kiyotoshi, Chiharu Ohta, Shigemitsu Ninomiya

**Affiliations:** ^1^ Department of Respiratory Medicine Toyokawa City Hospital Toyokawa City Japan; ^2^ Department of Respiratory Medicine, Allergy and Clinical Immunology Nagoya City University Graduate School of Medical Sciences Nagoya City Japan

**Keywords:** COVID‐19, cytokine, interstitial pneumonia, vaccine

## Abstract

Coronavirus disease 2019 (COVID‐19) has resulted in global morbidity, mortality, and societal disruption. COVID‐19 mRNA vaccination was mandatory in many countries for a period; however, severe adverse events have been observed in some individuals who received the vaccine. We report a case of COVID‐19 mRNA vaccine‐induced pneumonitis in a male patient whose cytokine levels were analysed prior to admission. His serum IgE level was high (347 U/mL), and several cytokines were elevated: TNFα was 1.2 pg/mL, IL‐6 was 156 pg/mL, and IL‐8 was 75.0 pg/mL. The patient developed severe respiratory failure requiring ventilatory support but recovered after steroid pulse therapy.

## Graphical abstract

We report a case of COVID‐19 mRNA vaccine‐induced pneumonitis in a male patient whose cytokine levels were analysed prior to admission. We examined his serum cytokine levels: TNFα was 1.2 pg/mL, IL‐6 was 156 pg/mL, and IL‐8 was 75.0 pg/mL. The patient developed severe respiratory failure requiring ventilatory support but recovered after steroid pulse therapy.

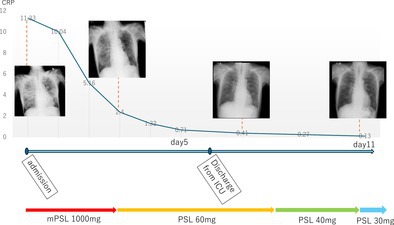

## 1 Introduction

Severe acute respiratory syndrome coronavirus 2 (SARS‐CoV‐2) has killed more than 14 million people [1]. The global pandemic and social disruption prompted the accelerated clinical application of COVID‐19 vaccines worldwide [2]. Since December 8, 2020, emergency use authorisation of mRNA vaccines (such as BNT162b2 (Pfizer‐BioNTech) and mRNA‐1273 (Moderna)) has been granted, and vaccination became compulsory in many countries. COVID‐19 mRNA vaccines are highly effective in preventing severe COVID‐19 and have contributed to pandemic control. However, the vaccines were rapidly approved, and unlike other investigational drugs, limited safety data had been accumulated. According to clinical trial data, most vaccine‐associated adverse events are mild [2]; however, severe adverse reactions such as anaphylaxis, myocarditis, and thrombotic events have also been reported [3]. These events can be fatal, and their mechanisms remain poorly understood. Pneumonitis following COVID‐19 vaccination is rare.

We report a case of severe pneumonitis as an adverse event of COVID‐19 vaccination. The patient recovered after steroid pulse therapy. His blood cytokine data before admission were examined. There have been few reports of cytokine analysis in acute vaccine‐related adverse events (AEs). We hope our report contributes to future cytokine research and therapy for vaccine‐related AEs.

## 2 Case Report

**FIGURE 1:**
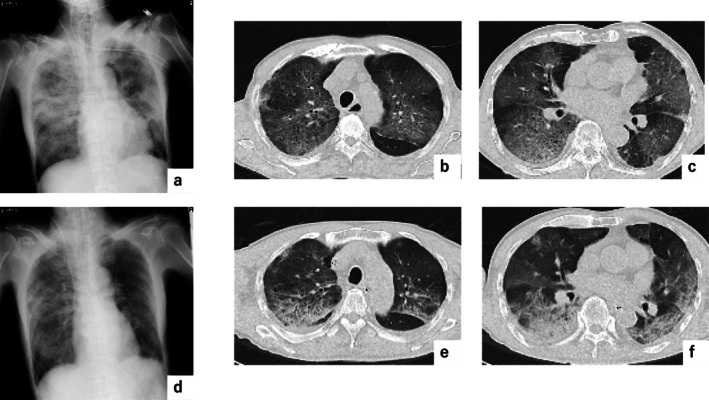
Chest x‐ray and CT findings on admission (a–c) and 2 days after admission (d–f).

A 77‐year‐old male ex‐smoker with a 30‐pack‐year history was admitted to our hospital with severe respiratory failure. He had no history of chronic interstitial lung disease and allergies. He had received his third dose of the COVID‐19 vaccine (BNT162b2, Pfizer) 2 days prior. Computed tomography (CT) revealed multiple ground‐glass opacities with traction bronchiectasis in both lungs (Figure 1A–C). On admission, his body temperature was 37.4°C, and oxygen saturation was 95% on nasal oxygen at 4 L/min. Significant pathogens were not detected and the quantitative test for COVID‐19 antigen was negative (Table 1). Chlamydophila pneumonia IgG and IgA were both positive, but the result indicated past infection (Table 1). His condition deteriorated in the emergency department, with oxygen saturation barely reaching 90% despite 10 L/min oxygen; therefore, invasive mechanical ventilation was initiated, and steroid pulse therapy was administered. As the aetiology of pneumonitis was unclear, antibiotic therapy (pazufloxacin, 1000 mg twice daily) was started under the suspicion of atypical pneumonia. The patient was admitted to the intensive care unit (ICU).

**TABLE 1:** Laboratory data of this case at admission.

Laboratory data	Value	Unit
Haematology		
White blood cell	9200	/μL
Neutrophils	82.1	%
Lymphocytes	10.7	%
Eosinophils	0.8	%
Basophils	0.1	%
Serology		
CRP	11.3	mg/dL
KL‐6	597	U/mL
BNP	242	pg/mL
Anti‐ARS Ab	< 5.0	index
β‐D‐glucan	4.4	pg/mL
ANA	< 40	fold
Total IgE	347	U/mL
Blood gas analysis (4 L/min)		
pH	7.495	
PaCO_2_	27.8	Torr
PaO_2_	60.0	Torr
$\mathrm{HC}O_{3}^{-}$	21.4	mmol/L
Cytokine		
IFNγ	< 0.1	IU/mL
IL‐1β	< 10	pg/mL
IL‐5	< 4	pg/mL
IL‐6	156	pg/mL
IL‐8	75.0	pg/mL
TNF‐α	1.2	pg/mL
Infection panel		
Quantitative COVID‐19 antigen	0.01 (Negative)	
Sputum bacterial culture	Negative	
Urinary pneumococcal antigen	Negative	
Urinary legionella antigen	Negative	
*C. pneumoniae* IgG (baseline data)	48 (94)	titre
*C. pneumoniae* IgA (baseline data)	16 (19)	titre

Abbreviations: ANA: antinuclear antibody; ARS: aminoacyl tRNA synthetase; BNP: brain natriuretic peptide; *C. pneumoniae* : *Chlamydophila pneumoniae* ; COVID‐19: coronavirus disease 2019; CRP: C‐reactive protein; IFN‐γ: interferon gamma; IgA: immunoglobulin A; IgG: immunoglobulin G; IL: interleukin; KL‐6: Krebs von den Lungen‐6; TNF: tumour necrosis factor.

**FIGURE 2:**
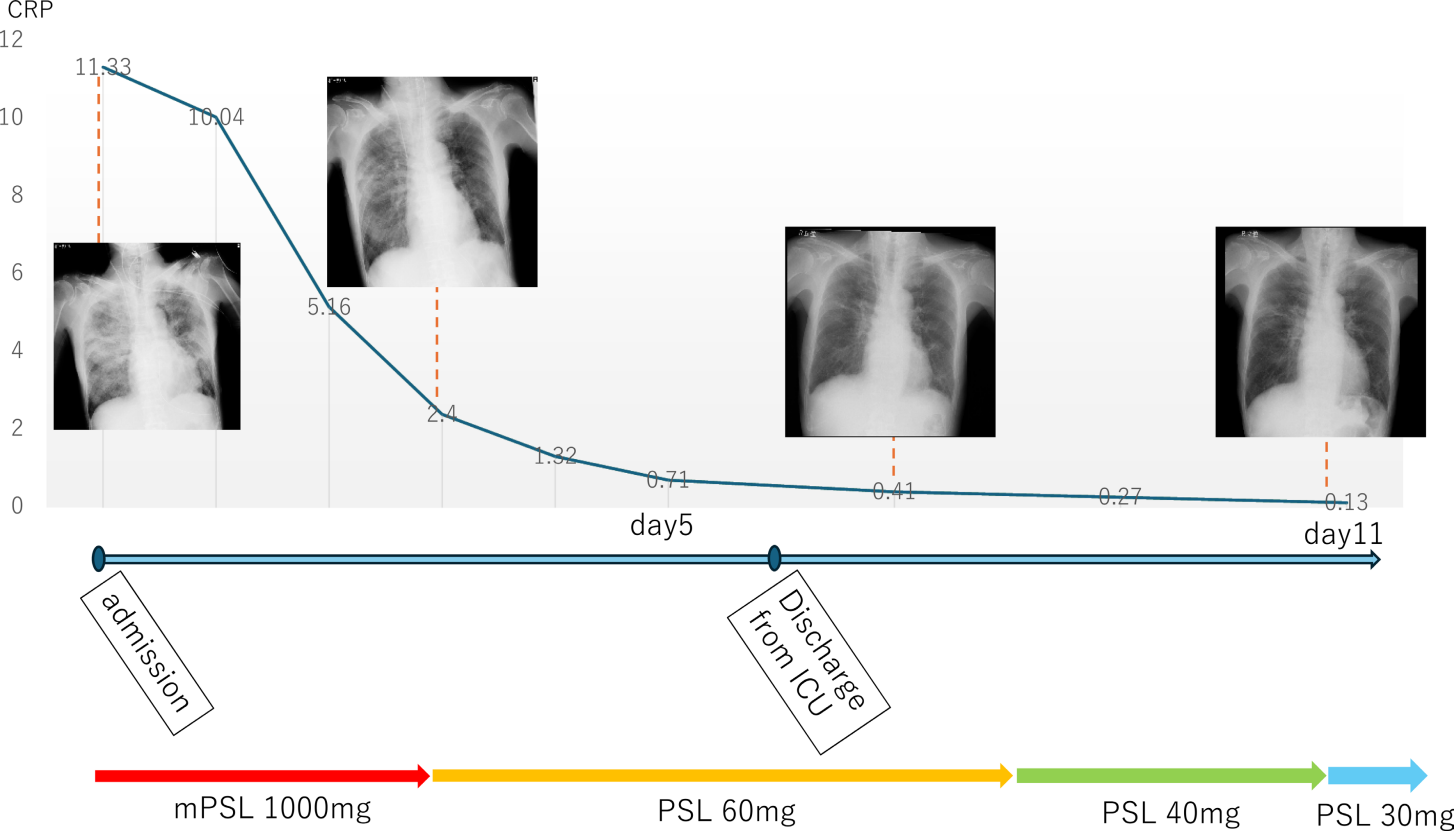
Timeline of chest x‐ray and CRP levels in relation to steroid therapy.

The patient received steroid pulse therapy for 4 days and recovered from respiratory failure. Antibiotic therapy was completed 5 days after admission. He was successfully weaned off ventilatory support and discharged from the ICU 6 days after admission. Corticosteroids were gradually tapered, and complete recovery was achieved within 2 weeks (Figure 2).

Pre‐admission laboratory tests (Table 1) showed a mildly elevated white blood cell count (9200/μL), with neutrophils at 82.1%, lymphocytes at 10.7%, and eosinophils at 0.8%. The serum KL‐6 level was normal, but C‐reactive protein (CRP) was elevated (11.3 mg/dL). All microbiological and immunological test results were negative.

Cytokine levels from pre‐admission serum were analysed (Table 1). IFN‐γ, IL‐1β, TNF‐α, and IL‐5 levels were within normal range, whereas IL‐6, IL‐8, and TNF‐α were elevated (TNF‐α: 1.2 pg/mL; IL‐6: 156 pg/mL; IL‐8: 75.0 pg/mL). IgE was slightly elevated (347 U/mL).

## 3 Discussion

In this case, the patient developed severe pneumonitis following COVID‐19 vaccination, and we evaluated cytokine dynamics during steroid therapy.

Analysis of circulating cytokines revealed markedly elevated IL‐8, IL‐6, and TNF‐α levels. In contrast, IL‐5, which plays a key role in allergic eosinophilic inflammation, was not elevated. However, the patient's IgE level was mildly elevated, suggesting a partial allergic response. Elevated IL‐8 has been reported in IgE‐mediated lung disease [4], supporting the possibility of an overlapping allergic component.

Meanwhile, IL‐6 and IL‐8 are also known mediators of systemic inflammation and cell‐mediated immune activation. Therefore, we speculate that this patient exhibited a mixed immune reaction—both allergic and inflammatory.

The mechanisms underlying allergic reactions to COVID‐19 vaccines remain poorly understood, and the number of documented cases is limited. Greenhawt et al. reported a very low incidence of vaccine‐associated anaphylaxis (7.91 per million doses) [5]. Lipid nanoparticles (LNPs), including polyethylene glycol and polysorbate, have been implicated in vaccine‐induced hypersensitivity and inflammatory reactions [5].

COVID‐19 mRNA vaccines encode the spike protein receptor‐binding domain (RBD) of SARS‐CoV‐2, prompting its expression on host cells to generate an immune response. The production of anti‐RBD antibodies not only protects against infection but may also enhance innate immunity. These antibodies may contribute to exaggerated immune responses, including systemic inflammation. Bergamaschi et al. reported that the second dose of BNT162b2 mRNA vaccine led to a sharp rise in cytokine levels, such as TNF‐α and IL‐6. Other studies have associated COVID‐19 vaccination with autoimmune manifestations and cytokine release syndrome. Together, these findings suggest that vaccination can stimulate various immune pathways, potentially leading to allergic‐like reactions and vaccine‐related pneumonitis.

In our case, cytokine assessment was limited by insurance restrictions in Japan, and bronchoscopy could not be performed due to rapid respiratory decline. Nevertheless, the observed elevations in cytokines and IgE support a vaccine‐related immune complication. Understanding these immunologic patterns may help guide treatment of future COVID‐19 vaccine‐related AEs.

## Author Contributions

**Tatsuro Suzuki:** investigation, writing, review, and editing. **Hiromi Furuta:** conceptualisation, investigation, writing, review, and editing. **Misa Naganawa** and **Keiko Hayashi:** investigation, review, and editing. **Hiroko Kiyotoshi:** review and editing. **Chiharu Ohta** and **Shigemitsu Ninomiya:** review, editing, and supervision.

## Consent

The authors declare that written informed consent was obtained for the publication of this manuscript and accompanying images using the consent form provided by the Journal.

## Conflicts of Interest

The authors declare no conflicts of interest.

## Data Availability

The data that support the findings of this study are available on request from the corresponding author. The data are not publicly available due to privacy or ethical restrictions.
